# Associations between breastfeeding duration and weight status transitions in early childhood

**DOI:** 10.1371/journal.pone.0323967

**Published:** 2025-05-29

**Authors:** Hyojun Park, Eric N. Reither

**Affiliations:** Department of Sociology and Anthropology, Utah State University, Logan, Utah, United States of America; School of Public Health, University of São Paulo, BRAZIL

## Abstract

Empirical evidence with respect to the protective effect of breastfeeding on childhood obesity remains inconclusive, and studies on sex-specific associations are sparse. We investigated whether (H1) longer breastfeeding duration reduces the risk of entry into elevated body mass (EBM); and (H2) longer breastfeeding duration increases the likelihood of exit from EBM. Using the Early Childhood Longitudinal Study, Birth Cohort (ECLS-B), our sample comprised 10,550 mother-child pairs after excluding 100 non-biological pairs. The ECLS-B assessed children from 9 months of age through kindergarten, spanning infancy through 7 years of age. We used two transitions in weight status (i.e., entry to EBM and exit from EBM) as outcome variables. The main predictor was breastfeeding duration (i.e., never breastfed, up to 2 months, 3 to 7 months, and 8 months or longer). Multilevel discrete-time models for recurrent transitions were employed to examine bidirectional changes in weight status. Among girls, breastfeeding for 8 months or longer reduced the risk of transitioning into overweight (adjusted Hazard Ratio (AHR): 0.76, 95% CI: 0.64, 0.89) or obesity (AHR: 0.66, 95% CI: 0.53, 0.82). Breastfeeding duration increased the likelihood of exiting EBM in a dose-response fashion among girls. Among boys, breastfeeding for 3 to 7 months was associated with preventing overweight (AHR: 0.79, 95% CI: 0.67, 0.92) or obesity (AHR: 0.82, 95% CI: 0.68, 0.98), but it did not help overcome EBM, regardless of breastfeeding duration. Breastfeeding duration helped to prevent EBM or overcome it, but these effects varied by the child’s sex. Further research should elucidate how the benefits of breastfeeding may differ for boys and girls, and explore the potential need for sex-specific public health policies.

## Introduction

Despite ongoing mitigation efforts, childhood obesity continues to pose significant challenges to healthcare providers and policymakers in the United States. In 2020, the prevalence of obesity among U.S. children aged 2 to 5 years reached 12.7% [1]. The consequences of childhood obesity are a major public health concern, as obesity is associated with increased risks of type 2 diabetes, autoimmune diseases, cardiovascular disease, and premature mortality. In addition, obesity can diminish quality of life by impairing cognitive function, cardiorespiratory fitness, and physical function across the life span [2].

Breastfeeding plays a crucial role in child growth and development, offering many benefits including optimal nutrition, immune protection, healthy growth, cognitive development, and enhanced mother-infant bonding [3–6]. These advantages extend beyond infancy, with breastfeeding linked to reduced risks of infections, obesity, and chronic diseases throughout life [7–9]. Recognizing these benefits, the American Academy of Pediatrics (AAP) and the World Health Organization (WHO) recommend exclusive breastfeeding for approximately the first six months, followed by continued breastfeeding with complementary foods for up to two years [5,10]. As of 2019, 55.8% and 35.9% of infants in the US were breastfed at 6 months and 12 months, respectively. Additionally, 45.3% and 24.9% were exclusively breastfed through 3 months and 6 months, respectively [11].

Breastfeeding promotes healthy infant growth by regulating food intake, delaying the introduction of solid foods, and enhancing gut health [12,13]. The protective effect of breastfeeding on childhood obesity has been widely studied, yet empirical evidence remains inconclusive [14–16]. For example, one meta-analysis revealed an inverse, dose-response association between breastfeeding duration and childhood obesity [17]. Furthermore, research suggests that breastfeeding can reduce a child’s chances of becoming obese by up to 25% [18]; infants who breastfeed for the first three months are less likely to overfeed, further reducing obesity risk [19]. Despite these intriguing findings, a recent systematic review concluded that there is insufficient evidence to support claims that breastfeeding duration is associated with childhood obesity [16].

Even less evidence exists on how breastfeeding-obesity associations may vary by sex. Most studies adjust for sex in their analyses but do not report sex-specific differences [16–20]. One cross-sectional study with retrospective questions found that exclusive breastfeeding for the first 6 months was inversely associated with obesity and percentage body fat at ages 4 to 8 years, regardless of sex [21]. Nevertheless, sex-specific associations between breastfeeding and obesity are plausible given documented sex differences in growth patterns during infancy and early childhood [22], breastfeeding and formula-feeding practices [23], and levels of attachment with offspring [24]. Limited evidence on this topic highlights the need for further research.

To address this gap in the literature, we investigated longitudinal associations between breastfeeding duration and subsequent transitions in overweight or obesity—and how these associations varied by sex. Specifically, we examined two hypotheses for girls and boys, separately: (H1) longer breastfeeding duration reduces the risk of entry into overweight or obesity; and (H2) longer breastfeeding duration increases the likelihood of exit from overweight or obesity. Our study employed a survival modeling framework using a nationally representative birth cohort in the United States. By exploring these associations for boys and girls using a prospective, nationally representative data source, our investigation may result in more tailored recommendations for breastfeeding practice in relation to obesity prevention strategies.

## Methods

### Data

We used data from the Early Childhood Longitudinal Study, Birth Cohort (ECLS-B), a nationally representative study administered by the National Center for Education Statistics (NCES) that provides comprehensive information on child growth and development from birth through kindergarten [24]. The target population of the ECLS-B is all children born in the United States in 2001 except for (1) children born to mothers less than 15 years of age or (2) children who died, were adopted, or moved permanently out of the U.S. before 9 months of data collection [24]. Our study population, followed from 2001 to 2008, is comprised of 10,550 mother-child pairs after excluding 100 infants whose mothers are not biologically related. The first wave, conducted in 2001–2002, assessed children at approximately 9 months of age, on average, with a range of 6–18 months. The second wave (2003–2004) assessed children at age 2 years, with a range of 22–26 months. By the third wave (2005–2006), children had reached preschool age (i.e., 4 years on average, with a range of 44–65 months). The fourth wave assessed children at kindergarten entry in 2006–2007 when they were typically between 5–6 years old (range 56–74 months). The fifth wave, a kindergarten follow-up conducted in 2007–2008, assessed children at ages 6–7 years (i.e., 70–86 months). The ECLS-B also included data from birth certificates of all infants. The response rates and sample sizes during the five waves were 74.1% (n = 10,700), 69.0% (n = 9,800), 63.1% (n = 8,950), 58.0% (n = 7,000), and 53.7% (n = 1,900), respectively, after adjusting for the previous wave’s response rates and the substitution of primary sampling units [25].

### Measures

We defined our outcome as a set of transitions in weight status for child participants across five waves of observation. These transitions were based on a series of body mass index (BMI = kg/m^2^) percentiles for children, which were derived from the measured weight and height of each child with corresponding sex- and age-specific BMI percentiles from the 2000 CDC growth charts [22]. Transitions in weight status included entry to elevated body mass (EBM) and exit from EBM, which can take place more than once for an individual during the study period. We assessed EBM transitions between (1) not overweight (i.e., BMI < 85^th^ percentile) and overweight (i.e., BMI ≥ 85^th^ percentile) and (2) not obese (i.e., BMI < 95^th^ percentile) and obese (i.e., BMI ≥ 95^th^ percentile) [26]. BMI percentiles were truncated at +3 of the highest and -3 of the lowest percentile values reported by the CDC [22,27,28].

Our time-invariant measure of breastfeeding was constructed from a series of questions at waves 1 and 2:

- “Did you ever breastfeed (the child)?”
- “Are you still breast-feeding (the child) now?”
- “For how many months did you breastfeed (the child)?”
- “How old was (child) in months when you completely stopped breast feeding him/her?”

From these questions, we categorized breastfeeding as never breastfed, up to 2 months, 3 to 7 months, and 8 months or longer. These categories of breastfeeding duration were chosen based on the empirical distribution (i.e., a roughly even number of mothers fit into the three different breastfeeding durations) as well as the categories used in previous studies [29–31]. We could not assess exclusive breastfeeding, which is defined as taking breastmilk only, excluding any other food or drink (even water) for the first 6 months of life [32–34], because the ECLS-B does not include a question about water intake.

We included age in months and its square as fixed and random effects to account for the non-linear nature of body mass change during infancy and early childhood [22,35]. We also included several potential confounders. Infant and maternal characteristics include race/ethnicity (i.e., non-Hispanic (NH) white, NH black, Hispanic, and others)), plurality (i.e., singletons vs. twins or multiples), delivery methods (i.e., vaginal vs. C-section), maternal age at birth (i.e., 15–19 y/o, 20–24 y/o, 25–29 y/o, 30–34 y/o, and 35 y/o or more), education level of mothers (i.e., less than high school, high school, and more than high school), marital status at birth (i.e., married vs. not married), and household income (i.e., 0-15K, 15-30K, 30-40K, 40-75K, and ≥75K). Maternal BMI before pregnancy was estimated using self-reported weight and height, and then classified as underweight (BMI < 18.5), normal (BMI between 18.5 and 24.9), overweight (BMI between 25 and 29.9), or obese (BMI ≥ 30) [36]. Maternal weight gain during pregnancy was obtained from the birth certificate and categorized as less than 7.2 kg, 7.3–14.0 kg, 14.1–20.8 kg, or 20.9 kg or more, per the empirical distribution of the measure.

### Statistical analyses

We employed multilevel discrete-time models to examine bidirectional transitions between non-EBM and EBM categories, simultaneously [37,38]. Given the design of ECLS-B, the precise time to event (e.g., from not obese to obese) could not be ascertained. However, the interval during which an event occurred is known, giving our analytic dataset a discrete time structure. At the same time, as up to four transitions during the five waves were nested within individuals, our dataset is multilevel. Finally, we right-censored observations when BMI was not determined due to non-response before the end of the study period.

Prior to analysis, we reconstructed the dataset as a set of stacked long-form person-wave files for each transition [37–40]. For example, in the first dataset we coded the transition from not overweight to overweight as 1, and no entry to overweight as 0. In the corresponding second dataset, we coded the transition from overweight to not overweight as 1, and no exit from overweight as 0. Using this dataset, we estimated the hazard of each type of EBM transition, using an identifier to distinguish EBM entry from EBM exit. Our analyses accounted for the potential correlation between transitions nested within individuals by including a random effect for each infant in the study [37–41]. We evaluated the robustness of our proportional hazards models by employing logistic regression, which is an alternative way of estimating discrete-time multilevel models with recurrent transitions [41–43].

Both crude and adjusted estimates of hazard ratios (and their confidence intervals) were produced in SAS to assess the two transitions in weight status by breastfeeding duration. We stratified analyses by infant’s sex to account for different growth patterns among boys and girls [22]. Using the survey weights provided by ECLS-B, we calculated robust standard errors (SE) (i.e., sandwich estimates) to account for unequal probabilities of selection and survey nonresponse [24]. Missing data in the analytic models, including censored transitions, were imputed 50 times using multiple imputations by chained equations (MICE) [44–47]. The robustness of our results was evaluated through additional analyses. First, instead of using a complementary log-log link function to estimate hazard ratios, we estimated odds ratios with a logit link function. Second, we conducted the analysis on an alternate study population that excluded twins or multiples (n = 1,750) and infants without BMI percentiles (n = 700).

Analyses were conducted using SAS version 9.4 (Cary, NC) and R (R Core Team). The data were accessed for research purposes on July 10, 2024. We rounded the number of cases reported in our manuscript to the nearest 50, per NCES guidelines [24]. Restricted data for the analyses were obtained with permission from the Institute of Education Sciences – NCES (Contract number: 21020012). Our university’s institutional review board considered this study exempt from review (protocol number: 12933).

## Results

Table 1 presents baseline characteristics of our study population by duration of breastfeeding for girls and boys, separately. Among girls, lower BMI percentiles were observed at longer breastfeeding durations. Conversely, no such association was observed among boys. For both girls and boys, breastfeeding was notably less common and shorter in duration among NH Black infants compared to infants of other races/ethnicities. Longer breastfeeding durations were observed among infants who were singletons and born via vaginal delivery. Infants tended to breastfeed for longer durations when their mothers were aged 25 or older, had more than a high school education, were married, had a household income of $40,000 or more, had a normal weight before pregnancy, and gained 16–46 pounds during pregnancy.

**Table 1:** Baseline characteristics of the study population by duration of breastfeeding and infants’ sex, ECLS-B.

	Girls					Boys				
	Duration of breastfeeding				p*	Duration of breastfeeding				*p* ^*^
	Never breastfed (n = 1,650)	up to 2 months (n = 1,250)	3 to 7 months (n = 1,150)	≥ 8 months (n = 1,100)		Never breastfed (n = 1,750)	up to 2 months (n = 1,300)	3 to 7 months (n = 1,200)	≥ 8 months (n = 1,100)	
Infant age (months)					0.35					<0.01
Mean	9.3	9.3	9.5	9.5		9.2	9.2	9.5	9.7	
(sd.)	(4.4)	(4.6)	(4.0)	(3.8)		(4.5)	(4.7)	(4.2)	(3.8)	
BMI percentiles					<0.01					0.45
Mean	57.0	60.0	58.4	52.2		60.0	60.9	61.2	59.3	
(sd.)	(31.9)	(31.5)	(31.0)	(31.3)		(32.5)	(31.5)	(30.0)	(31.7)	
Race/Ethnicity (%)					<0.01					<0.01
NH-White	39.4	40.0	39.1	45.5		40.0	42.3	41.7	45.5	
NH-Black	27.3	12.0	13.0	4.5		25.7	11.5	12.5	4.5	
Hispanic	18.2	24.0	21.7	22.7		17.1	23.1	20.8	22.7	
Other	18.2	20.0	26.1	27.3		17.1	23.1	25.0	27.3	
Plurality (%)					<0.01					<0.01
Singletons	87.9	84.0	87.0	90.9		77.1	76.9	79.2	90.9	
Twins or Multiples	18.2	20.0	17.4	9.1		17.1	19.2	16.7	9.1	
Delivery methods (%)					<0.01					<0.01
Vaginal delivery	63.6	64.0	65.2	72.7		60.0	61.5	70.8	72.7	
C-section	36.4	36.0	30.4	27.3		40.0	38.5	29.2	22.7	
Maternal age at birth (%)					<0.01					<0.01
15 - 19 y/o	15.2	16.0	8.7	4.5		17.1	15.4	8.3	4.5	
20 - 24 y/o	30.3	28.0	21.7	18.2		31.4	26.9	20.8	13.6	
25 - 29 y/o	24.2	24.0	26.1	27.3		22.9	23.1	25.0	27.3	
30 - 34 y/o	18.2	20.0	26.1	31.8		17.1	23.1	29.2	31.8	
35 y/o or more	12.1	12.0	17.4	22.7		11.4	11.5	16.7	22.7	
Education level of mothers (%)					<0.01					<0.01
Less than High school	18.2	12.0	8.7	9.1		17.1	11.5	8.3	9.1	
High school	51.5	40.0	30.4	22.7		51.4	38.5	29.2	18.2	
More than High school	30.3	44.0	56.5	68.2		31.4	46.2	62.5	68.2	
Maternal marital status (%)					<0.01					<0.01
Married	51.5	64.0	73.9	81.8		51.4	65.4	75.0	86.4	
Not married	48.5	36.0	26.1	18.2		48.6	38.5	25.0	13.6	
Household income (%)					<0.01					<0.01
0 - 15K	30.3	20.0	17.4	9.1		25.7	19.2	12.5	9.1	
15 - 30K	30.3	28.0	21.7	18.2		31.4	26.9	20.8	18.2	
30 - 40K	12.1	12.0	13.0	13.6		11.4	11.5	12.5	13.6	
40 - 75K	18.2	24.0	26.1	27.3		20.0	23.1	25.0	27.3	
75K -	9.1	16.0	26.1	31.8		11.4	19.2	29.2	31.8	
Maternal BMI before pregnancy (%)					<0.01					<0.01
Underweight	6.1	8.0	4.3	4.5		8.6	7.7	4.2	4.5	
Normal weight	51.5	52.0	56.5	59.1		48.6	53.8	62.5	63.6	
Overweight	21.2	20.0	21.7	22.7		22.9	23.1	20.8	18.2	
Obese	21.2	16.0	13.0	9.1		17.1	15.4	12.5	9.1	
Maternal weight gain during pregnancy (%)					<0.01					<0.01
less than 7.2 kg	18.2	12.0	13.0	9.1		14.3	11.5	8.3	4.5	
7.3 - 14.0 kg	33.3	32.0	34.8	31.8		34.3	30.8	33.3	31.8	
14.1 - 20.8 kg	21.2	28.0	21.7	27.3		22.9	23.1	25.0	27.3	
20.9 kg or more	12.1	12.0	8.7	9.1		11.4	11.5	12.5	9.1	

Data: Early Childhood Longitudinal Study, Birth Cohort (ECLS-B), National Center for Education Statistics (NCES)

Notes: 1. Unweighted statistics were presented to describe the sample. 2. Column percentages were presented for all variables except age and BMI. 3. The number of cases was rounded to the nearest 50 per the guideline of the NCES, and then used to obtain percentages. Percentages may not add up to 100%. 4. Information was missing for BMI percentiles (n = 700), duration of breastfeeding (n < 50), plurality (n = 100), delivery methods (n = 150), maternal age at birth (n = 100), education level of mothers (n = 250), maternal marital status (n = 100), maternal BMI before pregnancy (n = 250), and maternal weight gain during pregnancy (n = 2,250). * Differences between duration of breastfeeding categories were assessed using F-tests and chi-square tests.

**Abbreviations:** y/o (years old), BMI (body mass index), NH- (non-Hispanic), ≥ 8 months (8 months or longer)

Table 2 summarizes jointly estimated hazard ratios for the two transitions in weight status by breastfeeding duration among girls. In the crude model, relative to girls who were not breastfed, girls who breastfed for up to 2 months, 3–7 months, and 8 months or longer were less likely to transition into overweight status (BMI ≥ 85^th^ percentile) by 12% (hazard ratio (HR): 0.88, 95% CI: 0.76, 1.02), 16% (HR: 0.84, 95% CI: 0.71, 0.99), and 31% (HR: 0.69, 95% CI: 0.59, 0.80), respectively. At the same time, girls who breastfed for 8 months or longer were 50% (HR: 1.50, 95% CI: 0.1.26, 1.79) more likely than never-breastfed girls to exit from overweight. After adjusting for confounders, girls who breastfed for 8 months or longer were 24% (adjusted HR (AHR): 0.76, 95% CI: 0.64, 0.89) less likely to become overweight and 44% (AHR: 1.44, 95% CI: 1.19, 1.73) more likely to exit from overweight than never-breastfed girls. With respect to obesity (BMI ≥ 95^th^ percentile), girls who breastfed for 8 months or longer were 34% (AHR: 0.66, 95% CI: 0.53, 0.82) less likely to become obese and 58% (AHR: 1.58, 95% CI: 1.24, 2.02) more likely to exit from obesity than never-breastfed girls, after adjusting for confounders. Furthermore, we observed dose-response effects of breastfeeding on the likelihood of exiting obesity, with hazard ratios of 1.11 (95% CI: 0.89, 1.39), 1.36 (95% CI: 1.09, 1.71) and 1.58 (95% CI: 1.24, 2.02) for up to 2 months, 3–7 months, and 8 months or longer, respectively.

**Table 2:** Estimated hazard ratios for transitions in weight status by breastfeeding, Girls.

Breastfeeding	Weight Transitions	Crude Models			Adjusted Models		
		HR	LCI	UCI	AHR	LCI	UCI
	EBM: Overweight (BMI ≥ 85^th^ percentile)						
Never breastfed	Entry to EBM	1.00	(Reference)		1.00	(Reference)	
up to 2 months	Entry to EBM	0.88	0.76	1.02	0.90	0.78	1.05
3 to 7 months	Entry to EBM	0.84	0.71	0.99	0.91	0.77	1.07
8 months or longer	Entry to EBM	0.69	0.59	0.80	0.76	0.64	0.89
							
Never breastfed	Exit from EBM	1.00	(Reference)		1.00	(Reference)	
up to 2 months	Exit from EBM	0.97	0.81	1.16	0.96	0.80	1.16
3 to 7 months	Exit from EBM	1.17	1.00	1.37	1.13	0.95	1.34
8 months or longer	Exit from EBM	1.50	1.26	1.79	1.44	1.19	1.73
							
	EBM: Obese (BMI ≥ 95^th^ percentile)						
Never breastfed	Entry to EBM	1.00	(Reference)		1.00	(Reference)	
up to 2 months	Entry to EBM	0.80	0.66	0.98	0.83	0.68	1.02
3 to 7 months	Entry to EBM	0.87	0.72	1.05	0.96	0.78	1.17
8 months or longer	Entry to EBM	0.58	0.47	0.71	0.66	0.53	0.82
							
Never breastfed	Exit from EBM	1.00	(Reference)		1.00	(Reference)	
up to 2 months	Exit from EBM	1.11	0.90	1.37	1.11	0.89	1.39
3 to 7 months	Exit from EBM	1.34	1.09	1.65	1.36	1.09	1.71
8 months or longer	Exit from EBM	1.59	1.27	1.98	1.58	1.24	2.02

Data: Early Childhood Longitudinal Study, Birth Cohort (ECLS-B), National Center for Education Statistics (NCES)

Notes: 1. Outcomes were defined as two transitions in weight status, including entry to elevated body mass (EBM) (e.g., transition from non-obesity to obesity) and exit from EBM (e.g., transition from obesity to non-obesity) among the population at risk for each transition. 2. Crude models included breastfeeding duration, age, age-squared, and the time to transition; adjusted models additionally controlled for race/ethnicity, plurality, delivery methods, maternal age at birth, education level of mothers, maternal marital status, household income, maternal BMI before pregnancy, and maternal weight gain during pregnancy. 3. Presented models were fitted with a complementary log-log link function. Alternative models with a logit link function are available in S1 Table.

**Abbreviations:** HR (hazard ratio); AHR (adjusted hazard ratio); LCI (95% lower confidence interval); UCI (95% upper confidence interval); EBM (elevated body mass).

Table 3 presents jointly estimated hazard ratios for the two transitions in weight status by breastfeeding duration among boys. Relative to girls who breastfed from 3 to 7 months, strong protective effects were observed among boys who breastfed for this duration of time; such boys were 21% less likely to become overweight (AHR: 0.79, 95% CI: 0.67, 0.92) and 18% less likely to become obese (AHR: 0.82, 95% CI: 0.68, 0.98) than never-breastfed boys. However, among boys, breastfeeding for 8 months or longer did not reduce the hazard of becoming obese. Moreover, unlike girls, no duration of breastfeeding among boys was associated with a significantly increased likelihood of exiting from overweight or obesity status.

**Table 3:** Estimated hazard ratios for transitions in weight status by breastfeeding, Boys.

Breastfeeding	Weight Transitions	Crude Models			Adjusted Models		
		HR	LCI	UCI	AHR	LCI	UCI
	EBM: Overweight (BMI ≥ 85^th^ percentile)						
Never breastfed	Entry to EBM	1.00	(Reference)		1.00	(Reference)	
up to 2 months	Entry to EBM	1.00	0.87	1.15	1.00	0.86	1.16
3 to 7 months	Entry to EBM	0.75	0.65	0.88	0.79	0.67	0.92
8 months or longer	Entry to EBM	0.98	0.84	1.14	1.04	0.88	1.22
							
Never breastfed	Exit from EBM	1.00	(Reference)		1.00	(Reference)	
up to 2 months	Exit from EBM	1.02	0.87	1.20	1.01	0.85	1.19
3 to 7 months	Exit from EBM	1.10	0.92	1.32	1.07	0.89	1.30
8 months or longer	Exit from EBM	1.15	0.97	1.36	1.10	0.92	1.31
							
	EBM: Obese (BMI ≥ 95^th^ percentile)						
Never breastfed	Entry to EBM	1.00	(Reference)		1.00	(Reference)	
up to 2 months	Entry to EBM	0.96	0.81	1.14	0.98	0.83	1.16
3 to 7 months	Entry to EBM	0.76	0.64	0.90	0.82	0.68	0.98
8 months or longer	Entry to EBM	0.88	0.74	1.04	0.97	0.81	1.16
							
Never breastfed	Exit from EBM	1.00	(Reference)		1.00	(Reference)	
up to 2 months	Exit from EBM	1.13	0.95	1.35	1.13	0.94	1.35
3 to 7 months	Exit from EBM	1.15	0.95	1.39	1.14	0.94	1.38
8 months or longer	Exit from EBM	1.09	0.90	1.31	1.08	0.89	1.33

Data: Early Childhood Longitudinal Study, Birth Cohort (ECLS-B), National Center for Education Statistics (NCES)

Notes: 1. Outcomes were defined as two transitions in weight status, including entry to elevated body mass (EBM) (e.g., transition from non-obesity to obesity) and exit from EBM (e.g., transition from obesity to non-obesity) among the population at risk for each transition. 2. Crude models included breastfeeding duration, age, age-squared, and the time to transition; adjusted models additionally controlled for race/ethnicity, plurality, delivery methods, maternal age at birth, education level of mothers, maternal marital status, household income, maternal BMI before pregnancy, and maternal weight gain during pregnancy. 3. Presented models were fitted with a complementary log-log link function. Alternative models with a logit link function are available in S2 Table.

**Abbreviations:** HR (hazard ratio); AHR (adjusted hazard ratio); LCI (95% lower confidence interval); UCI (95% upper confidence interval); EBM (elevated body mass).

**Fig 1:**
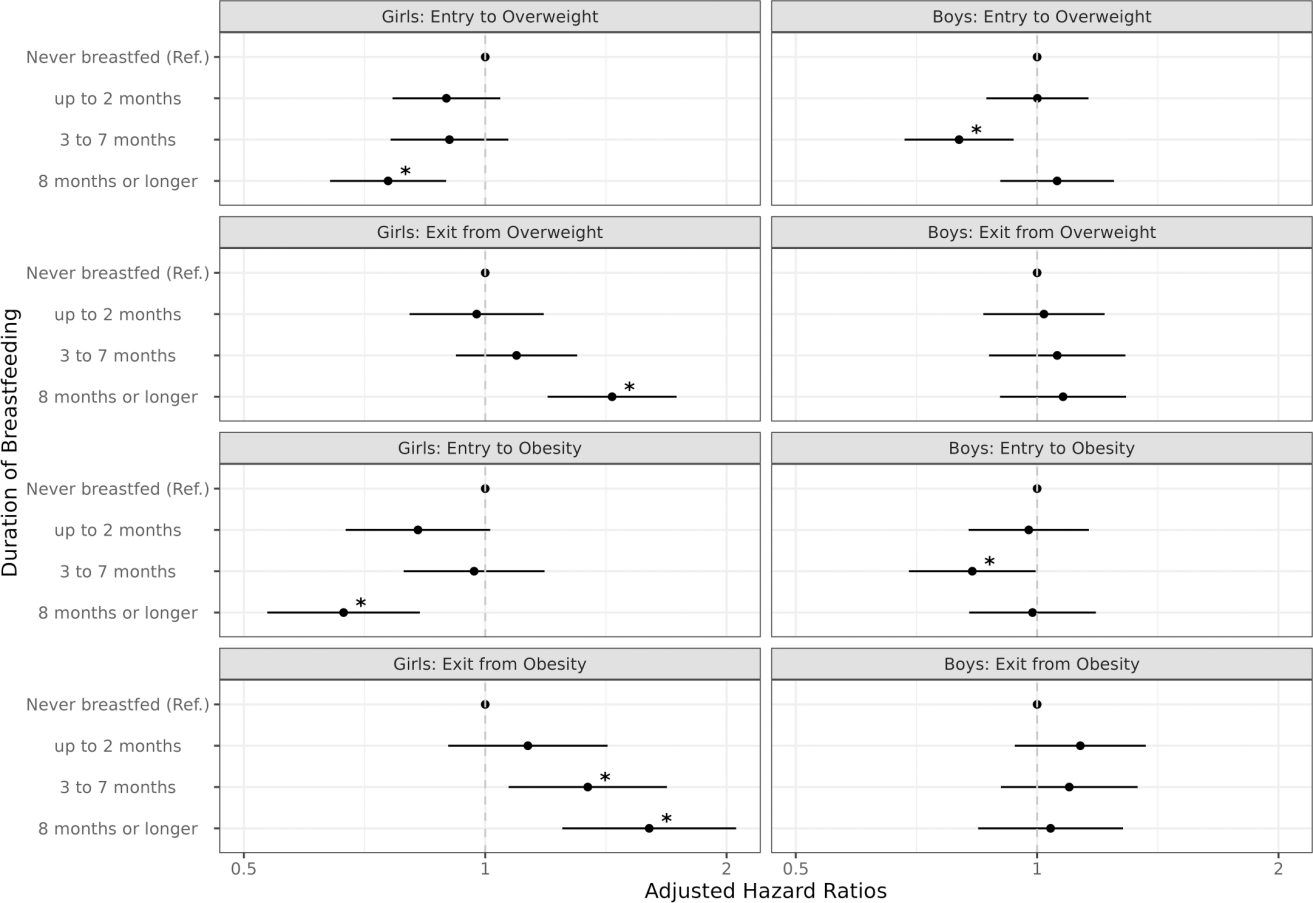
Comparisons of hazard ratios: Entry to vs. exit from EBM by infants’ sex. Data: Early Childhood Longitudinal Study, Birth Cohort (ECLS-B), National Center for Education Statistics (NCES) Notes: 1. Adjusted models with a complementary log-log link function were presented. 2. Outcomes were defined as two transitions in weight status, including entry to elevated body mass (EBM) (e.g., transition from non-obesity to obesity) and exit from EBM (e.g., transition from obesity to non-obesity) among the population at risk for each transition. 3. * significant at *p* < 0.05. (reference: never breastfed) **Abbreviations**: HR (hazard ratio); AHR (adjusted hazard ratio); LCI (95% lower confidence interval); UCI (95% upper confidence interval); EBM (elevated body mass).

As an aid to interpretation, the associations reported in Tables 2 and 3 are visualized in Fig 1. As noted, among girls there is a clear gradient effect between breastfeeding duration and the likelihood of exiting obesity. Hazard ratios for entry to overweight or obesity were significantly lower only among girls who breastfed for 8 months or longer. Among boys, hazards of becoming overweight or obese were significantly lower with 3–7 months of breastfeeding. These results were robust to a different model specification with a logit link function instead of a complementary log-log function (S1 and S2 Tables), as well as an alternate study population that excluded twins or multiples and infants without BMI percentiles (S3 and S4 Tables).

## Discussion

Using a nationally representative birth cohort in the U.S., we found that breastfeeding helped prevent and overcome EBM, but these effects varied by the child’s sex. Among girls, breastfeeding for 8 months or longer significantly reduced the risk of transitioning into overweight or obesity. We also observed a gradient effect in overcoming EBM among girls, where breastfeeding duration increased the likelihood of exiting overweight or obesity in a dose-response fashion. Among boys, breastfeeding for 3–7 months significantly reduced the hazard of transitioning into EBM, but it did not help overcome EBM, regardless of breastfeeding duration.

These findings provide partial support for our two study hypotheses. With respect to H1, we did not observe clear gradient effects in the association between breastfeeding duration and entry into EBM for either girls or boys. Nevertheless, we did find that relatively long breastfeeding durations reduced the hazards of becoming overweight or obese. For instance, girls who breastfed for 8 months or longer were 34% less likely to become obese than never-breastfed girls. Moreover, boys who breastfed from 3–7 months were 18% less likely to become obese than never-breastfed boys. With respect to H2, we found strong support among girls; there was a distinct gradient effect between breastfeeding duration and improved chances of exiting EBM. For example, the likelihoods of exiting from obesity were 11%, 36%, and 58% higher among girls who breastfed for up to 2 months, 3–7 months, and 8 months or longer, respectively, than among never-breastfed girls. Among boys, however, we found no support for H2; breastfeeding for any duration was not associated with the likelihood of exiting EBM.

Although our findings assess breastfeeding (not exclusive breastfeeding), they nevertheless generally align with professional recommendations, which promote exclusive breastfeeding during the first 6 months of life [5,10,48]. In addition, our findings suggest that relatively long breastfeeding durations may help prevent the onset of overweight or obesity. Breastfeeding confers physiological benefits that may reduce the risk of childhood obesity by providing essential nutrients and bioactive components that help regulate energy balance, metabolism, and fat deposition [6,49,50]. The nutritional and immunological properties of human milk likely contribute to healthier weight trajectories and insulin profiles in children, helping to prevent excessive weight gain [5]. These benefits provide a sound rationale for early intervention strategies that encourage breastfeeding during the first year of life to improve nutrition and potentially help combat childhood obesity [51].

As noted, findings in this study highlight the possibility that the protective effects of breastfeeding are somewhat different for boys and girls. Interestingly, prior research indicates that mothers’ milk composition and nutritional and hormonal requirements of infants may differ by infants’ sex [52]. For example, whereas mothers’ milk was higher in fat and protein with male infants, it had higher glucose concentrations with female infants [53]; such differences could reflect the fact that male infants tend to have higher daily energy requirements than female infants [54]. Infants’ sex may also influence breastfeeding practices. Prior research suggests that female infants tend to be breastfed for longer durations than male infants [55]; however, male infants may consume more breast milk per day than female infants [56]. Regarding other feeding practices, mothers tend to consume more energy dense foods with boys than girls from infancy through age 3 years [57], which could have sex-specific implications for breastfeeding-obesity associations. These physiological and behavioral differences between boys and girls are plausible mechanisms that could explain the sex-specific associations we observed between breastfeeding duration and EBM transitions. However, given limited research in this area, these sex-specific mechanisms must currently be regarded as speculative.

Sex-specific benefits of breastfeeding duration with respect to obesity prevention and weight loss necessitate both general and specific intervention strategies [58,59]. For example, while we support current breastfeeding recommendations for all infants, we also believe that girls may require longer breastfeeding durations to maximize its protective effects with respect to childhood obesity. Implementing breastfeeding intervention strategies will likely require a multidisciplinary approach, involving individual parent considerations, practitioner influences, hospital resources, societal factors (such as workplace and parental leave policies), access to lactation facilities, and social support [60]. The rise of commercial milk formula over the past century has posed a significant barrier to previous breastfeeding initiatives [61–63], requiring additional government-level interventions such as public health campaigns. Because physiological pathways are still debated and often not modifiable, interventions should target behavioral aspects of breastfeeding, including infant care and feeding practices during early childhood. In the absence of sex-specific recommendations from trusted organizations such as the WHO and AAP, further research is needed to build upon our finding that, while breastfeeding benefits boys and girls, it does so in different ways.

Consistent with our descriptive findings, studies have shown that certain sociodemographic subpopulations exhibit relatively low rates of breastfeeding. For instance, in 2020 the breastfeeding initiation rate was 74.5% among NH-Blacks, compared to 85.9% among NH-Whites and 86.8% among Hispanics [64]; the duration of breastfeeding was also shorter among NH-Blacks than other racial and ethnic groups [65]. Breastfeeding initiation rates also vary by socioeconomic status. Mothers with higher family incomes, higher education levels (either their own or their partner’s), and professional occupations are more likely than less fortunate mothers to initiate breastfeeding [66]. These disparities underscore the need for targeted interventions to support breastfeeding among vulnerable subpopulations that may experience barriers to optimal health practices.

There are a few important limitations of this study. First, we were unable to evaluate the effect of exclusive breastfeeding due to the way this variable is measured in the ECLS-B. Second, residual confounding from unobserved genetic and obesogenic environmental factors during infancy may have influenced our observed associations between the duration of breastfeeding and EBM transitions. For instance, feeding practices (e.g., food choices, timing, and quantity) and styles (e.g., how food is provided to infants), as well as food insecurity within families and communities, can significantly influence how mothers feed their offspring during infancy and early childhood. These factors, in turn, may be associated with both the duration of breastfeeding and EBM transitions [67,68]. Third, although our study is based on a nationally representative sample of infants, the findings may nevertheless not generalize to more recent birth cohorts.

Despite these limitations, this study has made significant contributions to our understanding of the associations between breastfeeding and obesity risk. Through the careful longitudinal analysis of a nationally representative birth cohort in the U.S., we have discovered sex-specific associations between breastfeeding duration and the hazards of EBM transitions. Our innovative analytical approach enabled us to distinguish between two critical pathways that affect overweight and obesity during childhood: entry into and exit from EBM. This distinction revealed different associations for boys and girls, pointing to underlying processes that likely differ in a sex-specific manner for each type of transition. Clearly, such mechanisms require further research to be understood adequately; nevertheless, our findings provide an important starting point for the further exploration of sex-specific processes that link breastfeeding to EBM transitions. By attempting to replicate and expand upon our findings, researchers could yield new insights for public health stakeholders as they develop targeted breastfeeding interventions.

## Conclusions

In summary, our study investigated longitudinal associations between breastfeeding duration and subsequent transitions in overweight or obesity, and how these associations varied by sex. We found that breastfeeding offers significant health benefits in preventing and overcoming childhood obesity, especially among girls. These findings support breastfeeding promotion efforts as a means of addressing the challenging public health issue of childhood obesity. Findings also emphasize the need for further research that elucidates how the benefits of breastfeeding may differ for boys and girls, as well as the potential need for sex-specific public health policies.

## Supporting information

S1 Table Estimated odds ratios for transitions in weight status by breastfeeding, Girls. Data: Early Childhood Longitudinal Study, Birth Cohort (ECLS-B), National Center for Education Statistics (NCES) Notes: 1. Outcomes were defined as two transitions in weight status, including entry to elevated body mass (EBM) (e.g., transition from non-obesity to obesity) and exit from EBM (e.g., transition from obesity to non-obesity) among the population at risk for each transition. 2. Crude models included breastfeeding duration, age, age-squared, and the time to transition; adjusted models additionally controlled for race/ethnicity, plurality, delivery methods, maternal age at birth, education level of mothers, maternal marital status, household income, maternal BMI before pregnancy, and maternal weight gain during pregnancy. 3. Presented models were fitted with a logit link function. **Abbreviations:** OR (odds ratio); AOR (adjusted odds ratio); LCI (95% lower confidence interval); UCI (95% upper confidence interval); EBM (elevated body mass).
(DOCX)

S2 Table Estimated odds ratios for transitions in weight status by breastfeeding, Boys. Data: Early Childhood Longitudinal Study, Birth Cohort (ECLS-B), National Center for Education Statistics (NCES) Notes: 1. Outcomes were defined as two transitions in weight status, including entry to elevated body mass (EBM) (e.g., transition from non-obesity to obesity) and exit from EBM (e.g., transition from obesity to non-obesity) among the population at risk for each transition. 2. Crude models included breastfeeding duration, age, age-squared, and the time to transition; adjusted models additionally controlled for race/ethnicity, plurality, delivery methods, maternal age at birth, education level of mothers, maternal marital status, household income, maternal BMI before pregnancy, and maternal weight gain during pregnancy. 3. Presented models were fitted with a logit link function. **Abbreviations:** OR (odds ratio); AOR (adjusted odds ratio); LCI (95% lower confidence interval); UCI (95% upper confidence interval); EBM (elevated body mass).
(DOCX)

S3 Table Estimated hazard ratios for transitions in weight status by breastfeeding, Singleton Girls. Data: Early Childhood Longitudinal Study, Birth Cohort (ECLS-B), National Center for Education Statistics (NCES) Notes: 1. Twins or multiples (n = 1,750) and infants without BMI percentiles (n = 700) were excluded from the analyses. 2. Outcomes were defined as two transitions in weight status, including entry to elevated body mass (EBM) (e.g., transition from non-obesity to obesity) and exit from EBM (e.g., transition from obesity to non-obesity) among the population at risk for each transition. 3. Crude models included breastfeeding duration, age, age-squared, and the time to transition; adjusted models additionally controlled for race/ethnicity, delivery methods, maternal age at birth, education level of mothers, maternal marital status, household income, maternal BMI before pregnancy, and maternal weight gain during pregnancy. 4. Presented models were fitted with a complementary log-log link function. **Abbreviations:** HR (hazard ratio); AHR (adjusted hazard ratio); LCI (95% lower confidence interval); UCI (95% upper confidence interval); EBM (elevated body mass).
(DOCX)

S4 Table Estimated hazard ratios for transitions in weight status by breastfeeding, Singleton Boys. Data: Early Childhood Longitudinal Study, Birth Cohort (ECLS-B), National Center for Education Statistics (NCES) Notes: 1. Twins or multiples (n = 1,750) and infants without BMI percentiles (n = 700) were excluded from the analyses. 2. Outcomes were defined as two transitions in weight status, including entry to elevated body mass (EBM) (e.g., transition from non-obesity to obesity) and exit from EBM (e.g., transition from obesity to non-obesity) among the population at risk for each transition. 3. Crude models included breastfeeding duration, age, age-squared, and the time to transition; adjusted models additionally controlled for race/ethnicity, delivery methods, maternal age at birth, education level of mothers, maternal marital status, household income, maternal BMI before pregnancy, and maternal weight gain during pregnancy. 4. Presented models were fitted with a complementary log-log link function. **Abbreviations:** HR (hazard ratio); AHR (adjusted hazard ratio); LCI (95% lower confidence interval); UCI (95% upper confidence interval); EBM (elevated body mass).
(DOCX)
