# Supplementary material for: Associations between breastfeeding duration and weight status transitions in early childhood
Source: PLoS One. 2025 May 29;20(5):e0323967. doi: 10.1371/journal.pone.0323967 (PMC12121782; doi:10.1371/journal.pone.0323967)
Supplement: S1 Table — Data: Early Childhood Longitudinal Study, Birth Cohort (ECLS-B), National Center for Education Statistics (NCES) Notes: 1. Outcomes were defined as two transitions in weight status, including entry to elevated body mass (EBM) (e.g., transition from non-obesity to obesity) and exit from EBM (e.g., transition from obesity to non-obesity) among the population at risk for each transition. 2. Crude models included breastfeeding duration, age, age-squared, and the time to transition; adjusted models additionally controlled for race/ethnicity, plurality, delivery methods, maternal age at birth, education level of mothers, maternal marital status, household income, maternal BMI before pregnancy, and maternal weight gain during pregnancy. 3. Presented models were fitted with a logit link function. Abbreviations: OR (odds ratio); AOR (adjusted odds ratio); LCI (95% lower confidence interval); UCI (95% upper confidence interval); EBM (elevated body mass). (DOCX) [file pone.0323967.s001.docx]

**S1 Table. Estimated odds ratios for transitions in weight status by breastfeeding, Girls**

| Breastfeeding | Weight Transitions | Crude Models | | | Adjusted Models | | |
| --- | --- | --- | --- | --- | --- | --- | --- |
|  |  | OR | LCI | UCI | AOR | LCI | UCI |
|  | EBM: Overweight (BMI ≥ 85th percentile) | | | | | | |
| Never breastfed | Entry to EBM | 1.00 | (Reference) | | 1.00 | (Reference) | |
| up to 2 months | Entry to EBM | 0.86 | 0.73 | 1.02 | 0.89 | 0.75 | 1.06 |
| 3 to 7 months | Entry to EBM | 0.82 | 0.68 | 0.98 | 0.89 | 0.74 | 1.08 |
| 8 months or longer | Entry to EBM | 0.66 | 0.55 | 0.78 | 0.73 | 0.61 | 0.88 |
|  |  |  |  |  |  |  |  |
| Never breastfed | Exit from EBM | 1.00 | (Reference) | | 1.00 | (Reference) | |
| up to 2 months | Exit from EBM | 0.96 | 0.76 | 1.20 | 0.96 | 0.76 | 1.21 |
| 3 to 7 months | Exit from EBM | 1.21 | 0.99 | 1.48 | 1.17 | 0.94 | 1.46 |
| 8 months or longer | Exit from EBM | 1.70 | 1.35 | 2.14 | 1.62 | 1.27 | 2.06 |
|  |  |  |  |  |  |  |  |
|  | EBM: Obesity (BMI ≥ 95th percentile) | | | | | | |
| Never breastfed | Entry to EBM | 1.00 | (Reference) | | 1.00 | (Reference) | |
| up to 2 months | Entry to EBM | 0.80 | 0.65 | 0.98 | 0.82 | 0.66 | 1.02 |
| 3 to 7 months | Entry to EBM | 0.86 | 0.70 | 1.06 | 0.96 | 0.77 | 1.18 |
| 8 months or longer | Entry to EBM | 0.56 | 0.46 | 0.70 | 0.65 | 0.51 | 0.81 |
|  |  |  |  |  |  |  |  |
| Never breastfed | Exit from EBM | 1.00 | (Reference) | | 1.00 | (Reference) | |
| up to 2 months | Exit from EBM | 1.16 | 0.86 | 1.56 | 1.17 | 0.85 | 1.61 |
| 3 to 7 months | Exit from EBM | 1.50 | 1.11 | 2.03 | 1.54 | 1.11 | 2.15 |
| 8 months or longer | Exit from EBM | 1.99 | 1.41 | 2.80 | 1.97 | 1.36 | 2.86 |
